# Supplementary material for: Upper Respiratory Infection Drives Clinical Signs and Inflammatory Responses Following Heterologous Challenge of SARS-CoV-2 Variants of Concern in K18 Mice
Source: Viruses. 2023 Apr 11;15(4):946. doi: 10.3390/v15040946 (PMC10144791; doi:10.3390/v15040946)
Supplement: Supplementary file 1 [file viruses-15-00946-s001.zip › viruses-2303817-SI.pdf]

**Table S1. Non-synonymous (Ns) mutations of SARS-CoV-2 recovered from NT and lungs from mice infected or infected:challenged with WA1, Alpha or Delta.** To be included in the table, $N_s$ mutation were present at a frequency higher than 1% and at a minimum depth of 100 reads per nucleotide.

| $N_s$ mutation | No. obs. | Group | dpi/dpc | Tissue | $N_s$ mutation frequency (%) |
|---|---|---|---|---|---|
| ORF1a:A3697V | 2 | Alpha | 3 | lung | 24.44 |
| ORF1a:G82_M85delinsV | 4 | WA1 | 3 | lung | 1.1 - 1.97 |
| ORF1a:G82_M85delinsV | 1 | Alpha | 3 | lung | 3.68 |
| ORF1a:I222V | 2 | WA1 | 3 | lung | 1.04 - 1.78 |
| ORF1a:M85del | 1 | WA1 | 1 | lung | 1.88 |
| ORF1a:M85del | 3 | WA1 | 3 | lung | 1.04 - 1.99 |
| ORF1a:T1731I | 4 | WA1 | 3 | lung | 2.13 - 5.11 |
| ORF1b:D2175fs | 1 | Alpha | 3 | lung | 3.16 |
| ORF1b:D2175fs | 1 | Alpha | 1 | NT | 4.26 |
| ORF1b:L2497F | 2 | WA1 | 1 | lung | 2.1 - 2.26 |
| ORF1b:L2497F | 4 | WA1 | 3 | lung | 2.06 - 2.59 |
| ORF1b:L2497F | 3 | WA1 | 1 | NT | 4.24 - 7.01 |
| ORF1b:L2497F | 3 | WA1 | 3 | NT | 1.3 - 16.26 |
| ORF1b:L2497F | 2 | Delta | 1 | lung | 30-34 |
| ORF1b:L2497F | 2 | Delta | 3 | lung | 43.00 |
| ORF1b:L2497F | 3 | Delta | 1 | NT | 16 - 34 |
| ORF1b:L2497F | 2 | Delta | 3 | NT | 27 - 32 |
| ORF1b:K2395N | 1 | Delta | 1 | lung | 10.98 |
| ORF1b:K2395N | 2 | Delta | 3 | lung | 5.91 - 8.45 |
| ORF1b:K2395N | 4 | Delta | 1 | NT | 9.68 - 12.5 |
| ORF1b:K2395N | 1 | Delta | 3 | NT | 8.52 |
| S:N148K | 2 | WA1 | 1 | lung | 1.3 - 2.15 |
| S:N148K | 2 | WA1 | 3 | lung | 1.16 - 1.25 |
| S:N149K | 2 | WA1 | 1 | lung | 1.15 - 1.78 |
| S:N149K | 3 | WA1 | 3 | lung | 1.57 - 1.89 |
| S:N149K | 2 | WA1 | 1 | NT | 2.12 - 2.14 |
| S:N149K | 1 | WA1 | 3 | NT | 1.18 |
| S:N99delinsKLNY | 2 | WA1 | 1 | lung | 1.18 - 2.13 |
| S:N99delinsKLNY | 2 | WA1 | 3 | lung | 1.07 - 1.42 |
| S:N99delinsKLNY | 2 | WA1 | 1 | NT | 2.42 - 5.23 |
| S:N99delinsKLNY | 2 | WA1 | 3 | NT | 7 - 8.03 |
| S:D215_L216insKLRS | 3 | WA1 | 1 | lung | 7.39 - 11.22 |
| S:D215_L216insKLRS | 3 | WA1 | 3 | lung | 6.87 - 8.84 |
| S:D215_L216insKLRS | 4 | WA1 | 1 | NT | 5.17 - 11.46 |
| S:D215_L216insKLRS | 2 | WA1 | 3 | NT | 10.07 - 15.03 |
| S:D215N | 2 | WA1 | 1 | lung | 1.08 - 1.5 |
| S:D215N | 1 | WA1 | 3 | lung | 1.44 |
| S:D215H | 3 | WA1 | 1 | lung | 29.75 - 33.84 |
| S:D215H | 4 | WA1 | 3 | lung | 33.75 - 46.74 |
| S:D215H | 4 | WA1 | 1 | NT | 17.52 - 44.34 |
| S:D215H | 3 | WA1 | 3 | NT | 17.31 - 38.3 |
| S:E1072K | 2 | Delta | 3 | NT | 1.89 - 2.37 |

| | | | | | |
|---|---|---|---|---|---|
| S:E96A | 1 | WA1 | 1 | lung | 1.21 |
| S:E96A | 2 | WA1 | 3 | lung | 1 - 1.01 |
| S:V62G | 2 | WA1 | 1 | lung | 1.58 - 2.77 |
| S:V62G | 4 | WA1 | 3 | lung | 1.58 - 2.17 |
| S:V62G | 2 | WA1 | 1 | NT | 2.79 - 2.98 |
| S:V62G | 1 | Delta | 3 | lung | 4.39 |
| ORF3a:A39S | 1 | Alpha | 3 | NT | 2.48 |
| ORF3a:E239D | 1 | Alpha | 3 | lung | 1.02 |
| ORF3a:E239D | 1 | Alpha | 1 | NT | 1.94 |
| ORF3a:E239D | 1 | Alpha | 3 | NT | 3.47 |
| ORF3a:H182Y | 1 | Alpha | 1 | NT | 7.73 |
| ORF3a:S165Y | 1 | Alpha | 1 | NT | 1.61 |
| ORF3a:T176I | 1 | Alpha | 1 | NT | 1.92 |
| M:I24R | 1 | Alpha | 3 | NT | 2.29 |
| M:T7I | 3 | WA1 | 1 | lung | 32.86 - 39.47 |
| M:T7I | 4 | WA1 | 3 | lung | 41.24 - 47.04 |
| M:T7I | 4 | WA1 | 1 | NT | 23.22 - 53.16 |
| M:T7I | 3 | WA1 | 3 | NT | 28.36 - 43.36 |
| M:Y179fs | 1 | Alpha | 3 | NT | 1 |
| ORF6:I33T | 1 | Alpha | 3 | NT | 2.35 |
| ORF7a:*122L | 1 | Alpha | 1 | lung | 1.29 |
| ORF8:Q23H | 3 | Delta | 1 | lung | 1.44 - 1.88 |
| ORF8:Q23H | 4 | Delta | 3 | lung | 1.26 - 1.98 |
| ORF8:Q23H | 4 | Delta | 1 | NT | 1.42 - 3.59 |
| ORF8:Q23H | 4 | Delta | 3 | NT | 1.59 - 3.94 |
| ORF8:S67F | 1 | Alpha | 1 | NT | 1.4 |
| N:R14C | 1 | Alpha | 1 | NT | 1.43 |
| N:P365L | 1 | Alpha | 1 | NT | 3.57 |
| N:S194T | 1 | WA1:Alpha | 3 | NT | 98.02 |
| ORF10:L37F | 1 | Alpha | 1 | NT | 1.13 |

**Abbreviations**: nonsynonymous mutation ($N_s$), observations (obs.), days post-infection (dpi), days post-challenge (dpc)

**Table S2. Fold change of Log$_2$ values of selected RNA-Seq transcripts from lungs of infection or infection:challenge groups as compared to mock-inoculated on 1 dpi, 1 dpc, 3 dpi, or 3 dpc.** All values shown have a Log$_2$ fold change $\geq 1.5$ and a p value $\leq 0.05$.

| | **1 dpi** | | | **1 dpc** | | | **3 dpi** | | | **3 dpc** | | |
|---|---|---|---|---|---|---|---|---|---|---|---|---|
| ***Gene*** | **WA1** | **Alpha** | **Delta** | **WA1: WA1** | **WA1: Alpha** | **WA1: Delta** | **WA1** | **Alpha** | **Delta** | **WA1: WA1** | **WA1: Alpha** | **WA1: Delta** |
| *AICDA* | - | - | - | 3.38 | 2.94 | 4.75 | - | - | - | 3.86 | 3.66 | 3.72 |
| *C3AR1* | - | - | - | - | - | - | 4.15 | 2.91 | 2.67 | - | - | - |
| *CCL2* | 3.27 | 1.74 | 1.65 | - | - | 1.55 | 6.86 | 6.16 | 6.35 | - | - | - |
| *CCL3* | 1.80 | - | - | - | 1.54 | | 3.02 | 3.06 | 2.86 | - | - | - |
| *CD80* | - | - | - | - | - | - | 2.00 | 1.58 | 1.45 | - | - | - |
| *CD86* | - | - | - | - | - | - | 1.58 | 1.69 | 1.37 | - | - | - |
| *CD8A* | 3.01 | - | - | - | - | - | - | - | - | - | - | - |
| *CXCL10* | 6.10 | 3.72 | 4.25 | 2.91 | 2.82 | 3.97 | 7.59 | 7.85 | 7.81 | 2.10 | 2.22 | 2.30 |
| *CXCL11* | 6.33 | 3.19 | 3.50 | 3.61 | 3.39 | 4.57 | 8.26 | 8.57 | 8.15 | - | - | - |
| *DDX58* | - | - | - | - | - | - | 2.16 | 2.12 | 1.98 | - | - | - |
| *FCGR1* | 2.06 | 2.03 | 2.00 | - | - | - | 4.27 | 3.74 | 3.70 | - | - | - |
| *IDO1* | - | - | - | - | - | - | 2.73 | 2.75 | 2.63 | - | - | - |
| *IFIH1* | - | - | - | - | - | - | 2.38 | 2.38 | 2.10 | - | - | - |
| *IFNB1* | 4.54 | - | - | - | - | - | 8.95 | 8.39 | 6.84 | - | - | - |
| *IFNG* | - | - | - | - | - | 1.68 | 2.06 | 2.63 | 2.17 | - | - | - |
| *IL10* | - | - | - | - | 3.10 | 3.11 | 3.07 | 2.85 | 2.03 | - | - | - |
| *IL1RN* | - | - | - | - | - | - | 2.89 | 2.93 | 2.58 | - | - | - |
| *IL27* | - | - | - | - | - | - | 2.59 | 2.89 | 2.51 | - | - | - |
| *IL6* | 3.41 | - | - | - | - | - | 5.65 | 5.80 | 5.57 | - | - | - |
| *IRF7* | 3.06 | 3.00 | 2.68 | - | - | - | 5.05 | 4.88 | 4.80 | - | - | - |
| *JCHAIN* | - | - | - | 4.35 | 4.40 | 4.21 | - | - | - | 3.78 | 3.92 | 3.93 |
| *OAS2* | 2.22 | 2.15 | 1.92 | - | - | - | 2.95 | 2.81 | 2.66 | - | - | - |
| *OAS3* | 2.26 | 2.36 | 2.01 | - | - | - | 4.14 | 4.05 | 3.74 | - | - | - |
| *TLR2* | - | - | - | - | - | - | 1.60 | 1.54 | - | - | - | - |
| *TLR7* | - | - | - | - | - | - | 1.66 | - | - | - | - | - |
| *TNF* | - | - | - | - | - | 1.56 | 2.54 | 2.66 | 2.47 | - | - | - |
| *ZBP1* | 2.92 | 2.75 | 2.56 | - | - | - | 4.13 | 3.91 | 3.84 | - | - | - |

**Abbreviations:** days post-infection (dpi), days post-challenge (dpc), dash (-) represent any fold change less than 1.5 and/or a P value <0.05.

**Table S3. Fold change of $Log_2$ values of selected RNA-Seq transcripts from nasal turbinates from infection or infection:challenge groups as compared to mock-inoculated on 1 dpi, 1 dpc, 3 dpi, or 3 dpc.** All values shown have a $Log_2$ fold change $\geq 1.5$ and a p value $\leq 0.05$.

| | **1 dpi** | | | **1 dpc** | | | **3 dpi** | | | **3 dpc** | | |
|---|---|---|---|---|---|---|---|---|---|---|---|---|
| ***Gene*** | **WA1** | **Alpha** | **Delta** | **WA1: WA1** | **WA1: Alpha** | **WA1: Delta** | **WA1** | **Alpha** | **Delta** | **WA1: WA1** | **WA1: Alpha** | **WA1: Delta** |
| *C3AR1* | - | - | - | - | - | - | 1.78 | - | - | - | - | - |
| *CCL2* | 3.94 | 3.22 | 3.49 | - | - | 2.86 | 4.29 | - | - | - | - | - |
| *CCL22* | - | - | 1.56 | - | - | 1.53 | - | - | - | - | - | - |
| *CCL3* | 3.34 | 2.83 | 2.89 | - | - | - | 1.98 | - | - | - | - | - |
| *CCL5* | 3.47 | 2.39 | 2.61 | - | 2.32 | 2.92 | 3.02 | 2.84 | 2.95 | - | - | - |
| *CD80* | - | - | - | - | - | - | 1.56 | - | - | - | - | - |
| *CD8A* | 2.16 | 2.16 | 2.03 | 2.92 | 3.04 | 3.58 | 2.87 | 2.51 | 2.55 | 2.40 | 3.15 | 3.47 |
| *CXCL10* | 7.83 | 7.20 | 7.09 | - | 5.39 | 6.01 | 5.60 | 3.33 | 3.85 | - | - | - |
| *CXCL11* | 7.39 | 6.55 | 6.44 | - | 5.66 | 6.16 | 4.01 | - | - | - | - | - |
| *DDX58* | 3.06 | 3.00 | 2.83 | - | - | 1.62 | 1.78 | - | - | - | - | - |
| *FCGR1* | 2.72 | 2.30 | 2.35 | - | - | - | 2.70 | - | 2.18 | - | - | - |
| *IDO1* | - | - | - | - | - | 5.69 | 6.27 | - | - | - | - | - |
| *IFIH1* | 3.45 | 3.30 | 3.03 | - | 1.57 | 1.95 | 2.03 | 1.66 | 2.06 | - | - | - |
| *IFNB1* | 7.87 | 8.24 | 7.27 | - | - | - | 7.12 | - | - | - | - | - |
| *IFNG* | 4.94 | 4.28 | 4.17 | - | 4.46 | 5.54 | 4.54 | - | - | - | - | - |
| *IL15* | - | - | - | - | - | - | - | - | - | - | - | - |
| *IL27* | 3.60 | - | - | - | - | - | - | - | - | - | - | - |
| *IL6* | 7.77 | 6.85 | 6.59 | - | - | 4.49 | 4.14 | - | - | - | - | - |
| *IRF7* | 4.72 | 4.60 | 4.38 | - | 2.96 | 3.44 | 4.01 | 4.16 | 4.60 | - | - | - |
| *JCHAIN* | - | - | - | 1.81 | - | - | - | - | - | - | - | 2.71 |
| *NOS2* | 2.10 | - | - | - | - | - | - | - | - | - | - | - |
| *OAS2* | 4.32 | 4.42 | 4.09 | - | 3.13 | 3.46 | 2.89 | 2.80 | 3.19 | - | - | - |
| *OAS3* | 4.75 | 4.76 | 4.51 | - | 3.47 | 3.86 | 3.77 | 3.10 | 3.72 | - | - | - |
| *TLR2* | 1.50 | - | - | - | - | - | - | - | - | - | - | - |
| *TLR3* | 2.74 | 2.62 | 2.44 | - | - | - | - | - | - | - | - | - |
| *TLR7* | - | - | - | - | - | - | 2.52 | - | - | - | - | - |
| *TLR8* | - | - | - | - | - | - | 2.29 | - | - | - | - | - |
| *TNF* | 2.57 | - | - | - | - | - | - | - | - | - | - | - |
| *ISG15* | 4.86 | 4.88 | 4.59 | - | 3.27 | 3.52 | 3.12 | 3.39 | 3.90 | - | - | - |
| *ZBP1* | 4.85 | 4.68 | 4.56 | - | 3.57 | 4.28 | 3.60 | 3.35 | 3.86 | - | - | - |

**Abbreviations:** days post-infection (dpi), days post-challenge (dpc), dash (-) represent any fold change less than 1.5 and/or a P value $<0.05$.

**Table S4. Neutralizing titers of the pre- and post-challenge sera of mice against WA1, Alpha, or Delta antigen.**

| Mouse ID | Challenge strain | Antigen | 21 dpi | 21 dpc | Fold change |
|---|---|---|---|---|---|
| 117 | WA1 | WA1 | 2213.8 | 5654.0 | 2.6 |
| 118 | WA1 | WA1 | 937.2 | 2432.7 | 2.6 |
| 119 | WA1 | WA1 | 2211.4 | 1527.4 | 0.7 |
| 120 | WA1 | WA1 | 1895.6 | 2931.0 | 1.5 |
| 153 | Alpha | WA1 | 1042.7 | 1853.8 | 1.8 |
| 154 | Alpha | WA1 | 655.1 | 2425.2 | 3.7 |
| 155 | Alpha | WA1 | 904.2 | 1971.8 | 2.2 |
| 156 | Alpha | WA1 | 1166.0 | 1531.3 | 1.3 |
| 141 | Delta | WA1 | 1841.6 | 7272.8 | 3.9 |
| 142 | Delta | WA1 | 1554.7 | 3758.9 | 2.4 |
| 143 | Delta | WA1 | 3757.6 | 4213.8 | 1.1 |
| 144 | Delta | WA1 | 2924.3 | 3106.6 | 1.1 |
| 117 | WA1 | Alpha | 1224.0 | 4558.7 | 3.7 |
| 118 | WA1 | Alpha | 931.2 | 1364.6 | 1.5 |
| 119 | WA1 | Alpha | 1522.0 | 928.1 | 0.6 |
| 120 | WA1 | Alpha | 1171.4 | 1631.9 | 1.4 |
| 153 | Alpha | Alpha | 831.2 | 1188.3 | 1.4 |
| 154 | Alpha | Alpha | 558.2 | 2992.2 | 5.4 |
| 155 | Alpha | Alpha | 669.3 | 2261.5 | 3.4 |
| 156 | Alpha | Alpha | 560.6 | 2215.7 | 4.0 |
| 141 | Delta | Alpha | 1074.9 | 1670.4 | 1.6 |
| 142 | Delta | Alpha | 1639.8 | 3935.4 | 2.4 |
| 143 | Delta | Alpha | 1089.6 | 1288.0 | 1.2 |
| 144 | Delta | Alpha | 1751.5 | 1444.8 | 0.8 |
| 117 | WA1 | Delta | 246.1 | 2583.0 | 10.5 |
| 118 | WA1 | Delta | 123.0 | 835.4 | 6.8 |
| 119 | WA1 | Delta | 1711.0 | 457.2 | 0.3 |
| 120 | WA1 | Delta | 1776.1 | 1446.4 | 0.8 |
| 153 | Alpha | Delta | 156.0 | 515.9 | 3.3 |
| 154 | Alpha | Delta | 139.4 | 1650.6 | 11.8 |
| 155 | Alpha | Delta | 130.1 | 234.9 | 1.8 |
| 156 | Alpha | Delta | 320.5 | 738.0 | 2.3 |
| 141 | Delta | Delta | 262.2 | 543.6 | 2.1 |
| 142 | Delta | Delta | 332.8 | 1528.0 | 4.6 |

| | | | | | |
|---|---|---|---|---|---|
| 143 | Delta | Delta | 228.2 | 733.0 | 3.2 |
| 144 | Delta | Delta | 428.6 | 380.2 | 0.9 |

**Abbreviations:** days post-infection (dpi), days post-challenge (dpc).
